# Decrease of cocaine, but not heroin, self-administration and relapse by the tyrosine kinase inhibitor masitinib in male Sprague Dawley rats

**DOI:** 10.1007/s00213-018-4865-0

**Published:** 2018-03-08

**Authors:** A. Belin-Rauscent, J. Lacoste, O. Hermine, A. Moussy, B. J. Everitt, David Belin

**Affiliations:** 1 Department of Psychology, University of Cambridge, Downing Street, Cambridge, CB2 3EB UK; 2 Behavioural and Clinical Neuroscience Institute, University of Cambridge, Downing Street, Cambridge, CB2 3EB UK; 3 Department of Psychiatry & Addictology, CHU de Martinique, Fort de France, Martinique France; 4 AB Science, Paris, SA France; 5 Institut Imagine INSERM U1163 and CNRS ERL8654, Centre de Reference des Mastocytoses, University of Paris Descartes, Paris, France; 6 Department of Hematology, APHP, Necker Children’s Hospital, Paris, France

**Keywords:** Tyrosine kinase inhibitor, Mast cells, Cocaine, Heroin, Addiction

## Abstract

**Rationale:**

Accumulating evidence shows that cocaine, and also heroin, influence several tyrosine kinases, expressed in neurons and in non-neuronal populations such as microglia, astrocytes and mast-cells. Drug-induced activation of mast cells both triggers inflammatory processes in the brain mediated by the glial cells they activate, and facilitates histamine release which may directly influence the dopamine system. Thus, by triggering the activation and degranulation of mast cells dependent on the tyrosine kinase c-kit and Fyn, the latter being also involved in NMDA-dependent synaptic plasticity, cocaine and heroin may indirectly influence the neural mechanisms that mediate their reinforcing properties. Masitinib, a novel tyrosine kinase inhibitor with high selectivity for c-Kit, Fyn and Lyn, may alter the aberrant consequences of the activation of these tyrosine kinases by cocaine and heroin.

**Objective:**

We investigated in rats the effect of a chronic oral treatment with masitinib (20 mg/kg) on the reinforcing and motivational properties of self-administered cocaine (250 μg/infusion) and heroin (40 μg/infusion).

**Methods:**

Three different cohorts of rats were trained instrumentally to respond for cocaine, heroin or food under continuous reinforcement. In each group, we assessed the influence of chronic daily treatment with masitinib on the maintenance of instrumental responding and intake and the motivation for the reinforcer. Thus, masitinib and vehicle-treated rats were challenged to adapt to high behavioural demand, to respond under a progressive ratio schedule of reinforcement and to reinstate instrumental responding after extinction and/or abstinence.

**Results:**

Masitinib selectively decreased cocaine intake, the motivation for cocaine and the subsequent propensity to respond for cocaine under extinction, while having no effect on instrumental responding for heroin or food.

**Conclusion:**

The present findings suggest masitinib, a drug with proven efficacy in CNS disorders, could represent a novel treatment for cocaine addiction provided its influence on the reinforcing and incentive properties of the drug is confirmed.

## Introduction

Accumulating evidence shows that exposure to cocaine or heroin influences the function of several tyrosine kinases (Lee and Messing 2008; Nestler 1994) which are involved in the regulation of transduction mechanisms both in neurons and non-neuronal populations, thereby contributing to the regulation of synaptic homeostasis and plasticity, as well as inflammation.

In neurons, the src kinases, such as Fyn and Lyn, control synaptic mechanisms, including plasticity, downstream of the NMDA receptor (Hayashi et al. 1999). Fyn is involved in the cocaine-induced alteration of NMDA-mediated glutamatergic transmission in the ventral tegmental area or the dorsal hippocampus that underlies the sensitisation to the psychomotor properties of cocaine (Schumann et al. 2009) or context-induced reinstatement of an extinguished instrumental response for cocaine (Xie et al. 2013), respectively. Similarly, src kinases have been suggested functionally to converge with PKC in the regulation of NMDA receptors by μ-opiate receptors (Garzon et al. 2008), the phosphorylation of which by Fyn is also involved in heroin withdrawal (Zhang et al. 2017).

In non-neuronal populations, cocaine and heroin trigger the src kinase-dependent activation of microglia and astrocytes in the brain and the activation of mast cells (Galli et al. 1993), the latter being involved in the propagation of drug-induced peripheral inflammation (de Timary et al. 2017; Liang et al. 2016; Nevidimova et al. 2015) to the brain (Kousik et al. 2012; Lacagnina et al. 2017).

Exposure to alcohol, cocaine and heroin triggers activation of astrocytes and microglia (Miguel-Hidalgo 2009), especially in the striatum where astrocytes, through their regulation of glutamate homeostasis, have been shown in rats to play a pivotal role in the propensity to reinstate extinguished instrumental responding for cocaine and heroin (Knackstedt and Kalivas 2009; Scofield and Kalivas 2014) and compulsive relapse after escalated intake of cocaine (Ducret et al. 2016). The involvement of astrocytes in mediating the reinforcing effect of cocaine has been recently shown to be under the control of inflammatory processes (Northcutt et al. 2015; but see Skolnick et al. 2014 for further discussion), the systemic activation of which has also been suggested to increase striatal dopamine release in response to stimulant drugs (Petrulli et al. 2017).

The primary mechanism bridging peripheral and central inflammation relies on the c-kit tyrosine kinase-dependent activation of peripheral mast cells (Dubreuil et al. 2009) and their subsequent degranulation in the brain (Dong et al. 2017; Skaper et al. 2014; Zhang et al. 2016). Even though there are resident mast cells in the brain (Zhuang et al. 1996), which provide up to 50 % of the brain’s histamine (Goldschmidt et al. 1985), the activation of glial cells (Skaper et al. 2014) and associated neuroinflammation depends on mast cells from the periphery (Dong et al. 2014; Theoharides 1990). Following activation, mast cells from the periphery have the ability to cross the blood brain barrier (Nautiyal et al. 2008), the permeability of which they control (Zhuang et al. 1996), and rapidly invade the brain where, upon degranulation, they release mediators such as dopamine, serotonin and CRF alongside cytokines and histamine (Dong et al. 2014). Thus, by triggering c-kit and Fyn-dependent activation and degranulation of mast cells, cocaine and heroin influence the permeability of the BBR and facilitate the release of histamine (Brown et al. 2001; Di Bello et al. 1998; Mannaioni et al. 1996). This degranulation-induced histamine release may activate astrocytes by recruiting cAMP-dependent signalling (Agullo et al. 1990) and independently influence histaminergic control of the function of the mesolimbic dopamine system, thereby directly interacting with the reinforcing or incentive properties of addictive drugs (Banks et al. 2009; Brabant et al. 2006, 2009; Ellenbroek 2013; Masukawa et al. 1993).

Thus, the tyrosine kinases c-kit and Fyn, alongside other members of the src kinase family, play a major role in the within- and between-systems adaptations to chronic exposure to cocaine or heroin that may influence the development of addiction. However, the lack of well-tolerated selective inhibitors with limited side effects has hitherto prevented the investigation of the influence of their chronic inhibition on the reinforcing and incentive properties of cocaine and heroin.

Masitinib is an oral active tyrosine kinase inhibitor that potently targets a limited number of kinases including c-Kit, Fyn and Lyn, as well as platelet-derived growth factor receptors, thereby controlling the central effects of Fyn and Lyn, the permeability of the BBR and the activation and degranulation of mast cells (Dubreuil et al. 2009). Studies involving kinase inhibitor selectivity have shown that masitinib is one of the most selective kinase inhibitors under development (Anastassiadis et al. 2011), thereby limiting the potential for off-target effects. Accordingly, masitinib has been shown to be effective and safe to use in humans for the treatment of mast cell-related diseases such as severe mastocytosis (Lortholary et al. 2017), severe refractory asthma (Humbert et al. 2009) and rheumatoid arthritis (Tebib et al. 2009), as well as in stroke (Gagalo et al. 2015), Alzheimer’s disease (Piette et al. 2011), multiple sclerosis (Vermersch et al. 2012) and depression (Moura et al. 2011, 2012).

We therefore investigated the influence of chronic daily per os administration of masitinib on the reinforcing and motivational properties of cocaine (250 μg/infusion) and heroin (40 μg/infusion) as compared to food. Three different cohorts of rats were trained to self-administer either cocaine, heroin or food and challenged under specific behavioural conditions to assess the influence of chronic masitinib administration on their sensitivity to the reinforcing properties of, and their motivation for, the drugs, as well as their propensity to relapse.

## Materials and methods

### Subjects

Male Sprague Dawley rats (*n* = 44, Charles River Laboratoires, Arbresle, France) weighting approximately 290 g at the start of the experiment were housed two per cage under a reversed 12 h light/dark cycle (lights on 7:00 P.M.) at controlled room temperature (22 °C). One week before the start of the experiments, rats were placed on a restricted diet of 20 g/day/rat lab chow, sufficient to maintained body weight and growth throughout the experiment. Water was available ad libitum and food was delivered every day 1 h after daily experimental session. All experiments were conducted between 8:00 am and 6:00 pm. All housing and testing were in accordance with the European Community Directives (2010/63/EU) and were approved by local animal care and use committee.

**Fig. 1:**
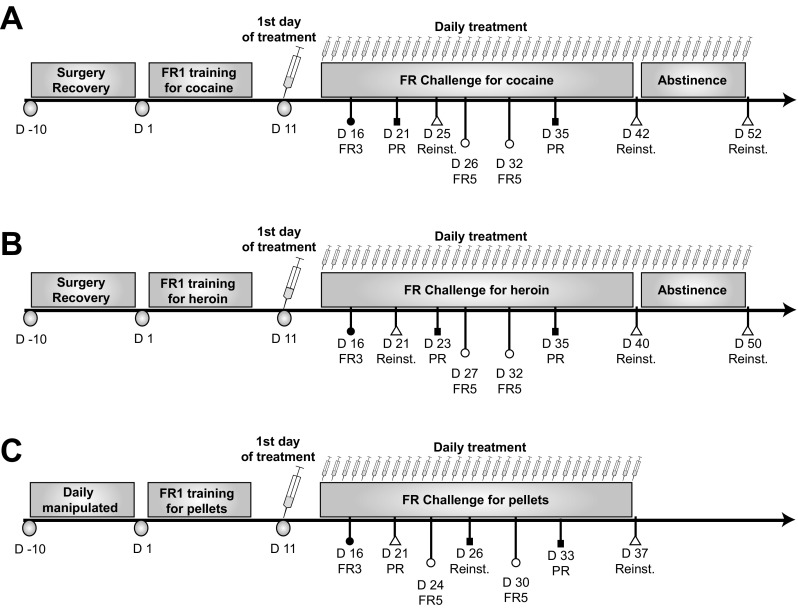
Timeline of the procedure for the cocaine (**a**), heroin (**b**) and food (**c**) self-administration groups. All Sprague Dawley male rats were habituated to the animal facility for a week during which they were handled and weighed daily. Subsequently, they were trained instrumentally to respond for cocaine, heroin or food pellets for 10 days under a fixed-ratio 1 schedule of reinforcement (FR1). Once stable performances were established (after 10 days), in each group, half of the population received daily oral treatment of masitinib (20 mg/kg) while the other half received vehicle (DMSO). Over the course of the training under masitinib treatment, animals were challenged to assess the reinforcing properties of the reinforcers by increasing the behavioural demand from FR1 to FR3: fixed-ratio 3 and FR5: fixed-ratio 5. Motivation for the drugs or food was measured under a highly challenging progressive ratio schedule of reinforcement (PR). The sensitivity to the incentive properties of the drug and the propensity to relapse were tested under extinction (Reinstatement) at different time points over the course of training/treatment and after 10 days of abstinence for the cocaine and heroin groups

Three experiments were performed in three separate groups of adult male Sprague Dawley rats trained instrumentally to respond for cocaine (*n* = 14), heroin (n = 14) or food (*n* = 12). Two rats died (1 cocaine, 1 heroin) as a result of surgical complications and two did not complete self-administration training because of catheter failure (1 cocaine vehicle-treated rat and 1 heroin masitinib-treated rat). The general timeline of the procedure is illustrated in Fig. 1.

### Treatment

Masitinib (AB1010; AB Science) was dissolved in DMSO which was also used as control vehicle. Masitinib (20 mg/kg) and vehicle were administered per os in 0.5 ml with a curved 80-mm cannula after a week of daily habituation to per os administration. Masitinib oral administration in the range of 15–25 mg/kg has been shown to yield plasma concentrations that are well above c-KIT IC50 of 150 nM, yet safe to use and effective at inhibiting mast cell activation and degranulation (Dubreuil et al. 2009). In a human phase 1 trial, the maximal tolerated dose of masitinib has not been reached in cancer patients who were orally administered up to 1000 mg/day, but Soria et al. (2009) concluded that 12 mg/kg/day can be considered as the maximal recommended dose for long-term treatment with masitinib. Since the doses in the range of 3 to 5 mg/kg are very well tolerated in humans and the rat equivalent doses of 18–30 mg/kg were shown to be equally effective and tolerated in rats (Barbeito 2017), the dose of 20 mg/kg was used in this study.

Because we were interested in testing the influence of the masitinib on well-established drug self-administration under conditions long well-characterised, treatment began only after 10 days of daily access to either cocaine, heroin or food, the unit doses of cocaine and heroin, namely 250 and 40 μg/infusion, respectively, being within the range of those with which a majority of self-administration studies are conducted in rats, including those using extended access and those having established compulsive drug intake or drug-seeking habits, reinstatement and inter-individual differences.

### Drug self-administration

#### Drugs

Heroin and cocaine (Cooperation Pharmaceutique, France) were dissolved in 0.9% sterile saline. Infusion doses were based on the hydrochloride salt form of each drug.

General procedures for cocaine and heroin self-administration are described in Fig. 1a, b.

#### Surgery

A single catheter (Camcath, UK, internal diameter = 0.28 mm; external diameter = 0.61 mm; dead volume = 12 μl) was implanted in the right jugular vein under ketamine hydrochloride (100 mg/kg, i.p.; Hippocampe, France)/xylazine (9 mg/kg, i.p.; Hippocampe, France) anaesthesia, according to a standardised procedure previously described (Vanhille et al. 2015). Briefly, the proximal end of the silastic catheter was inserted in the right atrium, and the distal end was sutured subcutaneously between the scapulae. To prevent infection, rats were treated with 10 mg/kg Baytril (s.c., Hyppocampe, France) the day before the surgery and during the first 6 days of the recovery. After surgery, catheters were flushed daily with a saline-heparin solution (100 IU/ml).

#### Apparatus

Self-administration experiments were conducted in 12 standard operant conditioning chambers (31.8 × 25.4 × 34.3 cm; Med Associates, St. Albans, VT) equipped with two 4-cm wide retractable levers, configured as previously described (Murray et al. 2013). The levers were 12 cm apart and 8 cm from the grid floor, and above each of them was a cue light (2.5 W, 24 V). A white house light (2.5 W, 24 V) was located on the top of the opposite wall. The floor of the chamber was covered with a metal grid with bars separated by 1 cm. The testing chamber was housed in a sound- and light-attenuating cubicle with a ventilation fan. In each chamber, a silastic tubing shielded with a metal spring extended from each animal’s intravenous catheter to a liquid swivel (Med Associates) mounted on an arm fixed outside the operant conditioning chambers. Tygon tubing extended from the swivel to a Razel infusion pump (Semat Technical, Herts, UK) located adjacent to the external chamber.

#### Acquisition of drug self-administration

Rats were trained to acquire cocaine (*n* = 14) or heroin (n = 14) self-administration in 2-h daily sessions under a fixed-ratio 1 (FR1) schedule of reinforcement during 10 days. Every active lever press resulted in a 20-s light presentation (conditioned stimulus, CS), an infusion of cocaine (250 μg/100 μl/5.7 s/infusion) or heroin (40 μg/100 μl/5.7 s/infusion) (McNamara et al. 2010), the extinction of the house light and retraction of both levers. Pressing on the inactive lever had no programmed consequence but was recorded to provide an index of general activity. During acquisition, the number of available infusions was limited to 60. Active and inactive lever assignment was counterbalanced and there was no lever-pressing training prior to drug SA, nor priming infusions.

### Instrumental conditioning with food

#### Apparatus

Food-reinforced instrumental conditioning took place in 12 operant conditioning chambers (31.8 × 25.4 × 34.3 cm, MedAssociates) equipped similarly to those used for drug self-administration. However, in this configuration, a food tray connected to a pellet dispenser was installed at the centre of the front wall. On each side of the food tray, two retractable levers were placed as described for self-administration.

General procedures for food reinforcement are presented Fig. 1c**.**

#### Magazine training and FR1 schedule of reinforcement for food

The day before the first session, rats (*n* = 12) were habituated to food pellets (45 mg dustless precision, Bioserv, Billaney) in their home cage to avoid any food neophobia. Then, rats were exposed to a 1 h magazine training session during which an average of 60 food pellets were delivered in a random interval schedule of reinforcement (RI 60 s).

Instrumental conditioning reinforced by food was initiated the day after the magazine training session. Each session started with the illumination of the houselight and insertion of the levers. Rats learned to lever press for pellets under an FR1 schedule of reinforcement. Each active lever press resulted in cue-light illumination above the lever (CS), a pellet distribution, extinction of the houselight and the retraction of the two levers. FR1 sessions lasted 30 min or until the rats earned 50 pellets, whichever occurred first. Presses on the inactive lever were recorded, but had no programmed consequence.

### Challenge procedures

#### Fixed-ratio challenge: FR3 and FR5 schedule of reinforcement

Reinforcing effects of the drugs or food were tested by increasing the behavioural demand for the same amount of reward (Spealman and Goldberg 1978).

#### Progressive ratio schedule

Motivation for the drug was measured by a highly challenging progressive ratio schedule of reinforcement (Belin and Deroche-Gamonet 2012; Ducret et al. 2016). The ratio of responses per infusion was increased after each infusion according to the following progression: 10, 20, 30, 45, 65, 85, 115, 145, 185, 225, 275, 325, 385, 445, 515, 585, 665, 745, 835, 925, 1025, 1125, 1235, 1345, 1465 and 1585. The maximal number of responses that a rat performed to obtain an infusion (the last ratio completed) is referred to as the breaking point (BP). The session ceased after either 5 h or when a period of 1 h elapsed since the previously earned infusion.

#### Persistence of responding under extinction/reinstatement

After about 2 weeks and a month of treatment, the effect of masitinib on persistence of responding in the absence of the drug was measured to assess as an additional proxy for the sensitivity to the incentive properties of the reinforcer the rats were trained to respond for (Belin et al. 2009). Thus, rats were challenged twice in 90-min extinction sessions in the absence of withdrawal. A similar test was performed after 10 days of forced abstinence from cocaine and heroin, in order to measure long-lasting propensity to resume responding for these drugs, so-called relapse. During these sessions, inactive lever presses had no consequences and rats rapidly extinguished their responding (in the first 10 to 20 min). Therefore, even if the entire session is presented, we only carried out statistical analyses (see below) on the first 15 min of each test (Rotge et al. 2017).

### Data and statistical analyses

Data are expressed as means ± SEM and were analysed using the StatSoft Statistica 10 package. Assumptions about normal distribution, homogeneity of variance and sphericity of the datasets were assessed using the Kolmogorov-Smirnov, Levene’s and Mauchly’s sphericity test, respectively. Data were then subjected to mixed model repeated measures analysis of variance (ANOVA). For the cocaine or heroin groups, between-subject differences were analysed on the acquisition and maintenance of self-administration by repeated measures ANOVAs with daily infusions as the dependent variables and the treatment group (masitinib vs vehicle) as between-subject factor. To assess the effect of masitinib treatment on motivation or reinstatement (as measured during the first 15 min of a 90 min long extinction session as previously described (Rotge et al. 2017), one-way repeated measure ANOVAs were carried out with active lever presses as dependent variable and treatment group (masitinib vs vehicle) as between-subject factor.

Upon confirmation of significant main effects, between-group differences were analysed using a Newman-Keuls or Dunnett post hoc tests, where appropriate. Non-parametric Kruskal-Wallis’s or Friedman’s test were conducted where appropriate for the food experimental group for which assumptions about normal distribution of the datasets were violated.

For all analyses, significance was set at *α* = 0.05. Partial eta-squared values (pη^2^) are reported as the measure of effect size to support the *p* values (Murray et al. 2015).

## Results

### Acquisition of instrumental learning in treated and control groups

**Fig. 2:**
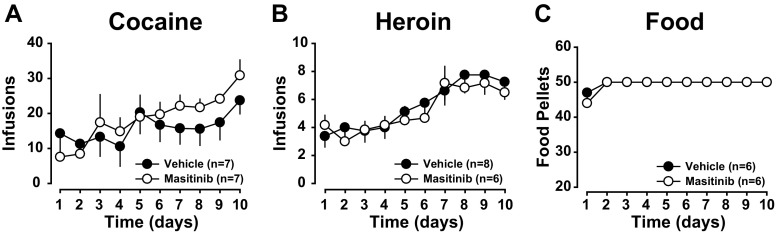
The experimental groups did not differ in their acquisition of instrumental responding for cocaine, heroin or food. All rats acquired cocaine (**a**) or heroin (**b**) self-administration within ten daily sessions. Similarly, rats trained to lever press for food (**c**) reached their maximal level of reinforcers earned by the second day of training. Moreover, prior to treatment, rats assigned to the masitinib group did not differ from those assigned to the control group in their intake of cocaine (**a**), heroin (**b**) or food **(c**)

All rats acquired cocaine (Fig. 2a) or heroin (Fig. 2b) self-administration within 10 days [main effect of session: F_9,108_ = 6.94, *p* < .0001, pη^2^ = .36 and F_9,108_ = 11.88, *p* < .0001, pη^2^ = .48 for cocaine and heroin, respectively]. Rats assigned to the masitinib group did not differ from those assigned to the control group as revealed by the lack of group × time interaction during the 10 days of acquisition [cocaine: main effect of group: F_1,12_ < 1, group × session interaction: F_9,108_ = 1.44, *p* = .18; heroin: main effect of group F_1,12_ < 1, group × session interaction: F_9,108_ < 1].

Similarly, rats trained to lever press for food earned their maximal amount of reinforcers as early as the second day of training (Fig. 2c). No differences were observed between rats assigned to the masitinib group and those assigned to the control group as revealed by the absence of difference between the mean rank of number of rewards earned for the 2 groups [ the Kruskall-Wallis test: all H_1, 12_ < 1].

### Masitinib selectively decreases cocaine intake

**Fig. 3:**
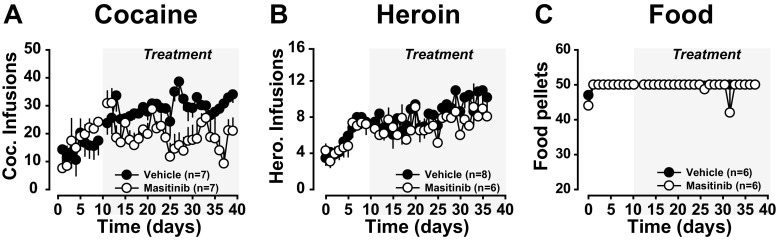
Masitinib selectively decreases the self-administration of cocaine at the unit dose of 250µg/infusion. After 2 weeks of daily training to self-administer either cocaine, heroin or food, rats were administered masitinib or its vehicle daily (grey panel). As compared to vehicle, masitinib treatment resulted in a marked and sustained reduction in cocaine intake (**a**). In marked contrast, masitinib treatment had no effect on the maintenance of heroin intake or the number of food pellets earned as compared to vehicle-treated rats (**b**–**c**)

After 10 days of training, daily masitinib treatment was initiated in half the population of rats responding for cocaine, heroin or food. On the first day of differential treatment, the cocaine groups (Veh vs masitinib) did not differ in their level of cocaine infusion (F_1,12_ < 1) (Fig. 3a). However, over the course of the 26 subsequent daily sessions, masitinib treatment resulted in a rapid and sustained decrease in cocaine intake as compared to vehicle treatment [main effect of treatment: F_1,12_ = 9.66, *p* < .01, pη^2^ = .44, treatment × session interaction F_25,300_ = 1.97, *p* < .01, pη^2^ = .14] (Fig. 3a).

In marked contrast, as compared to vehicle, masitinib had no effect on the number of heroin infusions received [main effect of treatment: F_1,12_ < 1, treatment × session interaction: F_21,252_ < 1] (Fig. 3b) or food pellets earned [ the Kruskall-Wallis test: all H_1, 12_ < 1] (Fig. 3c).

### Masitinib decreases the incentive properties of cocaine

**Fig. 4:**
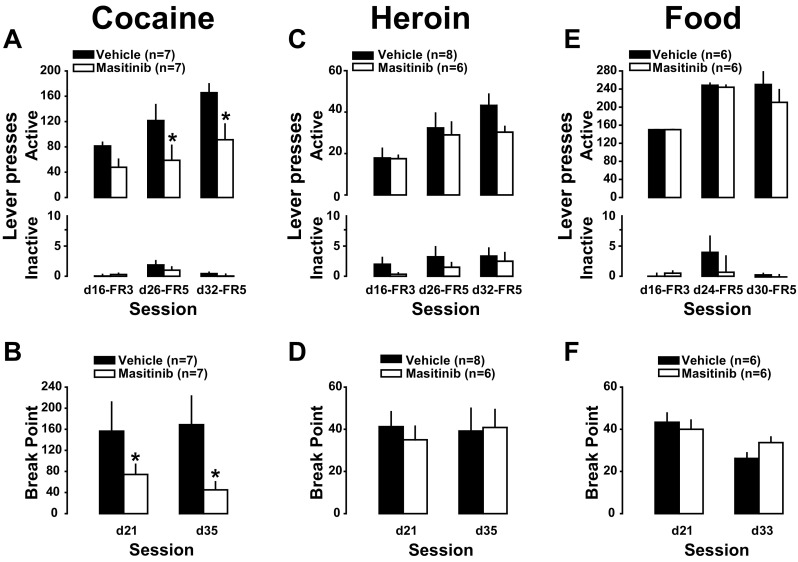
Masitinib decreases the incentive and motivational properties of cocaine at the unit dose of 0.25 mg/infusion. When the behavioural demand was increased from FR1 to FR3/FR5, vehicle-treated rats were able proportionally to increase their active lever presses in order to maintain a stable level of cocaine intake, an effort masitinib-treated rats were not capable of sustaining [**a**, * indicates *p* < .05 vs vehicle]. Similarly, the motivation for cocaine displayed by masitinib-treated rats under a progressive ratio schedule of reinforcement was markedly decreased as compared to the one displayed by vehicle-treated rats [**b,** * indicates *p* < .05]. In marked contrast, masitinib did not influence the increase in instrumental responding in the face of increased behavioural demand (**c** and **e**) or the motivation (**d** and **f**) for heroin or food. Masitinib had no effect on inactive lever presses across reinforcers (**a**, **c** and **e**)

We then investigated whether masitinib treatment also influenced the propensity of rats readily to adapt to increased behavioural demand (Salamone et al. 2007) to obtain their reinforcer. For this, masitinib- and vehicle-treated rats were challenged over the course of self-administration history with three sessions during which the behavioural demand was increased from FR1 to FR3 or FR5. As expected, these manipulations resulted in increase in instrumental responding, i.e., specific increase in active lever presses that parallels the increased behavioural demand in all groups [main lever × day interaction for cocaine: F_2,24_ = 9.1, *p* < .001, pη^2^ = .43, heroin: F_2,24_ = 11.93, *p* < .001, pη^2^ = .43 and food: Friedman’s test: *χ*^2^_12,2_ = 18.61, *p* < .001] (Fig. 4 top panel).

However, this ratio-dependent increase in instrumental responses was much higher in vehicle- than in masitinib-treated rats self-administering cocaine [main effect of treatment: F_1,12_ = 7.66, *p* < .02, pη^2^ = .39, lever: F_1,12_ = 81.857, *p* < .001 and treatment × lever interaction: F_1,12_ = 7.45, *p* < .02, pη^2^ = .38] (Fig. 4a). No effect of masitinib was observed in the rats self-administering heroin [main effect treatment: F_1,12_ = 1.21, *p* = .29, lever: F_1,12_ = 68.28, *p* < .001 and treatment × lever interaction: F_1,12_ < 1] (Fig. 4c) or food [ the Kruskall-Wallis test: all H_1, 12_ < 1] (Fig. 4e).

Additionally, masitinib-treated rats displayed a blunted motivation for cocaine as compared to vehicle-treated controls as revealed by lower break points during progressive ratio challenges performed 10 or 25 days after the initiation of treatment [main effect of treatment: F_1,12_ = 5.04, *p* < .05] (Fig. 4b). The effect of masitinib on motivation for cocaine was as effective after 10 days of treatment as it was after prolonged duration, i.e., 25 days, as revealed by a lack of effect of day or day × treatment interaction [Fs_1,12_ < 1].

In marked contrast, masitinib did not influence the motivation for heroin [main effect of treatment F_1,12_ < 1 and treatment × session interaction F_1,12_ < 1] (Fig. 4d) or food at any stage of treatment or self-administration history [the Kruskall-Wallis test for: PR1 and PR2: H_1,12_ < 1] (Fig. 4e).

### Masitinib decreases the persistence of responding for cocaine, but not heroin, under extinction tested both at early and late stages of training

**Fig. 5:**
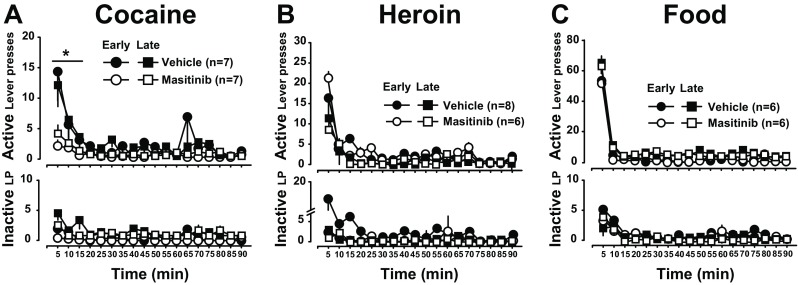
Masitinib reduces the persistence of instrumental responding for cocaine under extinction. Masitinib-treated rats displayed reduced persistence of cocaine seeking during extinction as measured as compared to the vehicle-treated animals at early and late stage of treatment. [**a**, * indicates *p* < .01 vs Veh for the first 15 min of extinction across sessions] For both heroin and food, masitinib did not influence lever pressing under extinction at early or late stage of treatment (**b** and **c)**

We further tested the influence of masitinib on the incentive properties of the reinforcers by measuring its effect on instrumental responding under extinction at various time points. Masitinib treatment greatly reduced the persistence of lever pressing for cocaine during both early and late extinction sessions carried out a day following a baseline SA session, e.g., with no preceding abstinence (Belin et al. 2009) on sessions 25 and 42. Thus, after either 2 weeks or a month of differential treatment, masitinib resulted in a decrease in active lever presses for cocaine [main effect of treatment: F_1,12_ = 6.58, *p* < .05, pη^2^ = .36, lever: F_1,12_ = 15.586, *p* < .01, treatment × lever interaction: F_1,12_ = 5.82, *p* < .05, pη^2^ = .32 but no effect of day or treatment × day interaction: F_1,12_ = 2.9, *p* = .12] (Fig. 5a). In contrast, masitinib did not influence responding under extinction for heroin [main effect of treatment, treatment × lever and treatment × day interaction: Fs_1,12_ < 1] (Fig. 5b) or food [ the Kruskall-Wallis test: all H_1,12_ < 1] (Fig. 5c).

### Masitinib treatment initiated in rats actively self-administering cocaine decreases subsequent relapse to cocaine seeking

**Fig. 6:**
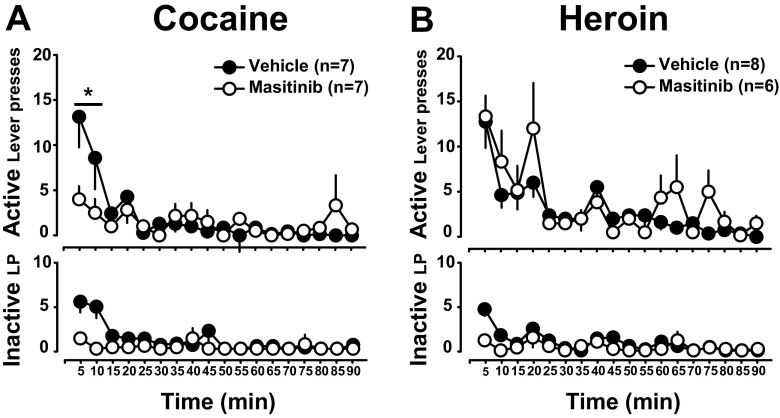
Masitinib decreases relapse to cocaine seeking. After 10 days of forced abstinence, masitinib-treated rats were less prone to relapse to instrumentally respond for cocaine as measured by active lever presses during the first 15 min of an extinction session [**a**, * indicates *p* < .01 for the first 10 min]. In marked contrast, after the same duration of forced abstinence from heroin, masitinib did not prevent relapse to heroin seeking (**b**)

Finally, we investigated whether masitinib influenced the propensity to relapse to seek cocaine or heroin after forced abstinence. As compared to vehicle controls, masitinib-treated rats displayed reduced relapse to instrumental responding for cocaine following 10 days of abstinence. Thus, masitinib-treated rats displayed a lower level of lever presses during the first 15 min of the reinstatement session as compared to vehicle-treated controls [main effect of treatment: F_1,11_ = 9.58, *p* < .02, pη^2^ = .46, lever: F_1,11_ = 7.97, *p* < .02 and treatment × lever interaction: F_1,11_ < 1] (Fig. 6a). Post hoc analyses revealed that masitinib-treated rats responded less on the active lever than the control group during the first 10 min of the extinction session (*p*s < .05). In marked contrast, masitinib had no effect on relapse to heroin seeking after 10 days of abstinence [main effect of treatment: F_1,12_ < 1, lever: F_1,12_ = 24.43, *p* < .001 and treatment × lever interaction: F_1,12_ = 1.6_,_ *p* = .23] (Fig. 6b).

## Discussion

The results of the present study demonstrate that daily treatment with the tyrosine kinase inhibitor masitinib results in a robust decrease in the reinforcing and motivational properties of cocaine in male rats with a relatively long history of self-administration of a unit dose of 250 μg/infusion. Thus, masitinib decreased cocaine intake under continuous reinforcement, prevented an increase in responding in the face of increasing behavioural demand for cocaine (Salamone et al. 2003) and decreased the break point under a progressive ratio schedule of reinforcement. This decrease in the motivation for cocaine in masitinib-treated rats was further supported by a marked decrease in the persistence of responding under extinction and the propensity to relapse after forced abstinence.

The effect of masitinib on instrumental responding was highly specific to cocaine at the dose tested as the same treatment had no effect on the reinforcing and motivational effects of food and heroin at the dose of 40 μg/infusion. The absence of effect on the motivation for food is in agreement with the overall lack of effect of masitinib in humans on feeding or general motivation. Thus, masitinib was recently shown to have potential therapeutic effects in depression in patients with mastocytosis (Moura et al. 2011, 2012), as well as in Alzheimer’s disease (Piette et al. 2011) and multiple sclerosis in humans (Vermersch et al. 2012), thereby demonstrating that this drug is safe to use.

Much to our surprise, masitinib had no effect on the reinforcing and motivational properties of heroin, suggesting that selective inhibition of c-kit, Fyn and Lyn exerted in the nanomolar range by masitinib only impinged on brain mechanisms of reinforcement engaged by cocaine in male rats.

In the light of the available research, it is difficult to put forward a definitive mechanism of the effect of masitinib. However, the specific behavioural effects and putative therapeutic potential of masitinib reported here warrant further research on the cellular mechanisms by which it exerts these effects.

Even though masitinib has high affinity for the microglial factor CSFR1, involved in microglia survival and activation, the differential influence of masitinib on the reinforcing and incentive properties of cocaine and heroin is very unlikely to be accounted for by a direct influence on central inflammatory mechanisms. Indeed, N-acetylcysteine (NAC) (Murray et al. 2012a), which acts centrally to prevent drug-induced neuroinflammation (Schneider et al. 2017) and directly targets astrocytes (Olive et al. 2012), does not show cocaine-specific effects as it decreases both cocaine and heroin-seeking behaviour (Hodebourg et al. 2018; Murray et al. 2012b). The specificity of the effects of masitinib is therefore more likely related to its effects on c-Kit, Fyn and Lyn.

Since neuronal Fyn and Lyn are involved in the regulation of NMDA-dependent synaptic mechanisms influenced by addictive drugs, including cocaine, alcohol and heroin (Ge et al. 2017; Schumann et al. 2009; Wang et al. 2010; Yaka et al. 2002), a direct influence of masitinib on Fyn may be a promising candidate mechanism. However, intracerebral inhibition of Fyn by the src kinase antagonist PP2 has been shown to inhibit heroin seeking as measured in a context-induced reinstatement procedure (Ge et al. 2017). Therefore, even if a direct influence on neuronal mechanisms cannot be ruled out, it seems unlikely to account for the differential effect of masitinib on the reinforcing and motivational properties of cocaine and heroin observed here.

However, Fyn and Lyn, alongside c-kit, primarily control the activation, migration and degranulation of mast cells, and therefore the mast cell glia axis (Zhang et al. 2016). Indeed, at the dose tested, masitinib is particularly efficient at inhibiting mast cells, thereby preventing drug-induced recruitment of neuroinflammatory mechanisms from the periphery (Di Bello et al. 1998; Dong et al. 2014; Petrulli et al. 2017; Silverman et al. 2000) and protecting against cocaine-induced alteration of the blood brain barrier (Kumar 2011; Sharma et al. 2009), the permeability of which is also controlled by mast cells (Esposito et al. 2002; Zhuang et al. 1996). However, the nature of the behavioural response to masitinib suggests it does not influence the brain availabilty of cocaine or heroin as such a difference would result in a upward vertical shift in responding under continuous reinforcement.

One potential alternative mechanism is related to the prevention by masitinib of drug-induced degranulation of activated mast cells having entered the brain. Thus, upon activation, these multifunctional cells enter the brain where alongside resident mast cells primarily, but not exclusively located in the thalamus (Dimitriadou et al. 1990; Goldschmidt et al. 1985; Zhuang et al. 1996), they release cytokines and neuromediators in their microenvironment. The neuromediators released by activated mast cells in the brain, include dopamine (in particular in the mesolimbic system; (Dropp 1976), corticotropin releasing factor, serotonin and histamine (Goldschmidt et al. 1985; Ronnberg et al. 2012a, b), which have all been shown to influence cocaine reinforcement (Silverman et al. 2000).

Histamine is of particular interest for the selectivity of the effects of masitinib on the motivational properties of cocaine as histaminergic mechanisms that influence the mesostriatal dopamine system (Banks et al. 2009; Ellenbroek 2013; Tanda et al. 2008). Although the nature of the interaction between the histamine and the dopamine system and consequent modulation of the reinforcing effects of cocaine remain to be elucidated (Banks et al. 2009; Brabant et al. 2009; Holtz et al. 2013; Ito et al. 1997; Oleson et al. 2012), histamine has been shown to act on different neuronal systems either to inhibit or activate midbrain dopamine activity (Fleckenstein et al. 1993; Molina-Hernandez et al. 2000; Schlicker et al. 1993).

Dopaminergic transmission in the mesolimbic system is a key mechanism underlying the reinforcing effects of cocaine, but less so heroin (Ettenberg et al. 1982; Pettit et al. 1984). Thus, heroin self-administration is unaffected by dopamine depletion from the nucleus accumbens, which in marked contrast reduces cocaine self-administration and progressive ratio break points for the drug (for review see Badiani et al. 2011). Since histamine brain levels are much more influenced by mast cells than the widespread projections from tuberomamillary nucleus of the hypothalamus, by inhibiting the degranulation of mast cells, masitinib may alter histaminergic control of dopaminergic mechanisms that underlie the reinforcing and motivational effects of cocaine, but not heroin. This clearly indicates the need for further investigation of the cellular mechanisms that mediate the effects of masitinib-induced degranulation of mast cells on the motivational effects of cocaine.

Additionally, in order fully to characterise the influence of masitinib on the reinforcing and motivational properties of cocaine, further investigations are required to test whether the effects observed here for the unit dose of 250 μg/infusion are generalised across a range of doses. Similarly, whether the decreased propensity instrumentally to respond under extinction after 10 days of forced abstinence results from a long-lasting influence of masitinib on previous cocaine self-administration or a direct effect on the latter should be further investigated.

Nevertheless, the present results suggest that a novel highly selective tyrosine kinase inhibitor that primarily targets mast cells activation and safe to use in humans decreases the reinforcing and motivational properties of 250 μg/infusion cocaine.
